# Cryo-EM of the injectisome and type III secretion systems

**DOI:** 10.1016/j.sbi.2022.102403

**Published:** 2022-08

**Authors:** Julien R.C. Bergeron, Thomas C. Marlovits

**Affiliations:** 1 Randall Centre for Cell and Molecular Biophysics, King's College London, London, UK; 2 Centre for Structural Systems Biology, University Medical Center Hamburg-Eppendorf, Hamburg, Germany

## Abstract

Double-membrane-spanning protein complexes, such as the T3SS, had long presented an intractable challenge for structural biology. As a consequence, until a few years ago, our molecular understanding of this fascinating complex was limited to composite models, consisting of structures of isolated domains, positioned within the overall complex. Most of the membrane-embedded components remained completely uncharacterized.

In recent years, the emergence of cryo-electron microscopy (cryo-EM) as a method for determining protein structures to high resolution, has be transformative to our capacity to understand the architecture of this complex, and its mechanism of substrate transport.

In this review, we summarize the recent structures of the various T3SS components, determined by cryo-EM, and highlight the regions of the complex that remain to be characterized. We also discuss the recent structural insights into the mechanism of effector transport through the T3SS. Finally, we highlight some of the challenges that remain to be tackled.

## Introduction

Cryo-EM as a method to characterize the structure of proteins was developed in the 1970's. However, for decades it remained a “niche” technique, mainly used to obtain low-resolution envelopes of large complexes that could not be crystallized (such as filaments, or double-membrane-spanning complexes) [1]. These were then used in conjunction with the crystal structures of isolated components, and other biochemical data, to propose atomic models of these complexes, a process often described as hybrid structural methods [2]. This however had major limitations, such as very inaccurate prediction of the protein/protein interfaces or discrepancies between structural and biochemical data, and was ultimately very unsatisfactory.

However, since the development of direct electron detectors, around 2014, and the associated “resolution revolution” [3], cryo-EM routinely allows to obtain the structure of protein complexes to near-atomic resolution. Following improvement in automation and computation also permits to solve multiple structures of heterologous and/or dynamic complexes [4], and to obtain structural insights for these within cells [5]. Collectively, these have transformed our capacity to understand large, complex, dynamic assemblies at the atomic level.

The injectisome is a large, megadalton-sized macromolecular assembly found at the surface of many gram-negative bacteria, and responsible for the injection of toxins (known as effector proteins) inside host cells during infection [6]. It therefore represents a major target for the development of new antimicrobial therapeutics [7]. It also possesses a striking syringe shape, reminiscent of its function, which has rendered the injectisome an object of fascination for researchers and the wider public alike. It employs a so-called type 3 secretion system (T3SS) machinery, also present in the bacterial flagellum, to export proteins across the cell membranes [8]. For simplicity, here we will employ the term T3SS to describe both the secretion apparatus and injectisome.

The existence of a T3SS was identified in the early 1990s [9], in multiple bacteria [10], and its syringe-shaped secretion apparatus, often referred to as the needle complex, was first isolated and imaged by negative-stain EM in 1998, in *Salmonella enterica* [11]. Following this, the first cryo-EM structure of the complex was reported 6 years later, to ∼17 Å resolution [11]. While a tour-de-force at the time, this structure only provided an envelope of the needle complex, revealing its overall architecture but very little about the arrangements of proteins within it. What could, however, be illustrated even at low resolution, were structural changes associated with different functional states. Seven years later, the T3SS structure was refined further to ∼10 Å resolution, and allowed to elucidate the oligomeric state of some of its components, and the outline of individual domains [12]. In parallel, the crystal structures of multiple components were solved in isolation; most of them were monomeric, but combined with the EM maps, they allowed to generate hybrid atomic models of increasing accuracy [13, 14∗, 15∗]. Nonetheless, these hybrid structural models were the state-of-the-art in the T3SS structural characterization, in 2014.

**Figure 1:**
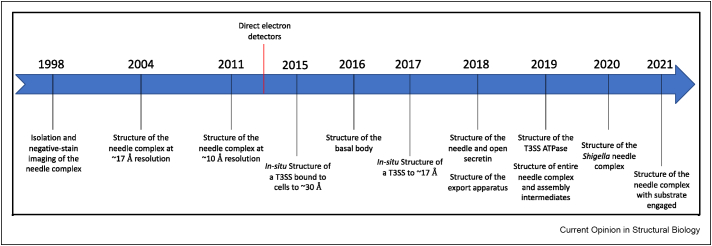
Timeline of the major cryo-EM studies revealing the T3SS structure and secretion mechanism.

Since then, the emergence of direct electron detectors, and the associated capacity to determine protein structures to near-atomic resolution by single-particle cryo-EM [3] has led to an explosion of studies, revealing the architecture of the needle complex and the cytoplasmic elements of the T3SS, as well as of the intact complex *in situ* (See timeline in Figure 1).

The aim of this review is to provide an overview of our understanding of the T3SS secretion apparatus structure, revealed by the cryo-EM structures obtained in the past ∼6 years. We will also discuss the aspects of the complex that remains to be elucidated, notably the translocon, as well as the details of the arrangement of the cytosolic components. Finally, we will discuss our current understanding of the mechanism of effector selection and secretion through the needle complex.

## Architecture of the T3SS secretion apparatus

**Figure 2:**
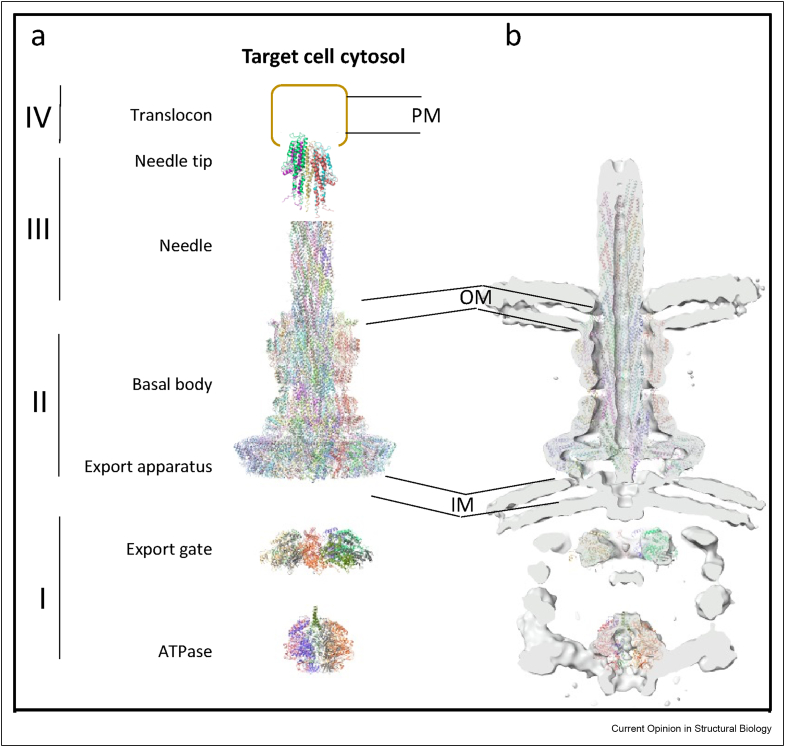
**Structure of the T3SS. (a)** Most recent structures of the NC complex, export gate, and ATPase, determined by cryo-EM. Components of unknown structure are shown in shapes. **(b)** Cryo-electron tomography map of the intact complex, with the corresponding atomic models fitted in.

The T3SS secretion apparatus can be divided into four regions (Figure 2): (I) On the cytoplasmic side, the ATPase and export gate are involved in the recruitment of effector proteins, and their transport through the apparatus; (II) a sub-complex termed the basal body spans both membranes and the periplasmic space; (III) on the extracellular side, the basal body is topped by the needle, a long (up to ∼ 100 nm, depending on the bacterial species) hollow filament, capped at the distal end by the needle tip; (IV) finally, the translocon complex forms a pore in the membrane of target cells, onto which the needle tip docks, activating the secretion of effectors, and their passage into the cytosol of the targeted cells [16].

a) Structure of the needle complex

When the T3SS was first identified, it rapidly became apparent that some of its components shared sequence similarity to the bacterial flagellum [17]. Following a purification protocol similar to that of the flagellum led to the isolation of a sub-complex consisting of the basal body and needle, termed needle complex (NC) [11]. Subsequent cryo-EM analysis, performed prior to the resolution revolution, and limited to ∼20 Å, provided an overall architecture of the complex and structural changes associated with different assembly states, but did not permit to identify specific protein components or domains [18]. In particular, the stoichiometry of the three basal body proteins, the inner-membrane proteins SctJ and SctD, and the outer-membrane protein SctC, remained elusive. A following cryo-EM analysis of the same NC complex reached sub-nanometer resolution [12], and importantly, established a 24-fold stoichiometry for the inner-membrane components, and surprisingly, 15-fold symmetry for the outer-membrane component. This was particularly surprising as multiple previous low-resolution cryo-EM studies of homologous proteins, belonging to the secretin family of outer-membrane proteins, had proposed a range of oligomeric states, from 12- to 16-mers, for this protein family [19, 20, 21]. Importantly, while the resolution of this later map did not permit to build the structure of its components *de novo*, it allowed to convincingly dock the crystal structures of individual domains determined in isolation, providing the first set of atomic models of the basal body [15,22].

Following the introduction of direct-electron detectors, the structure of the intact basal body (from a construct lacking the needle) was obtained to near-atomic resolution [23] (Figure 2a). This allowed the *de-novo* model building of the inner-membrane and outer-membrane complexes, and notably that of the long-coveted secretin domain. This structure confirmed its components’ stoichiometry, and provided the molecular details of their interfaces.

The T3SS needle consists of multiple repeats of a single protein, SctF. Similar to the basal body, early low-resolution cryo-EM studies provided insights in its overall architecture [24,25], but not the molecular details of the molecular arrangements within the complex. Hybrid methods combining this low-resolution EM data with solid-state NMR and modeling, allowed to construct atomistic models, sometimes contradictory, illustrating the challenges of such approaches [26,27]. However, with the emergence of direct-electron detectors, the structure of the T3SS filament was rapidly determined to near-atomic resolution, attached to the NC complex [28,29] (Figure 2a), providing the molecular details of secretin opening and interaction with the needle.

b) The export apparatus

On the cytosolic side, and embedded in the basal body on the inner-membrane side, a set of proteins (SctRSTU, collectively termed the export apparatus) are involved in the threading of unfolded effector proteins through the complex. The structure of these proteins, also conserved in the bacterial flagellum, had long remained a mystery, with only the crystal structure of the SctU soluble domain being determined [30,31]. However, since the cryo-EM resolution revolution, the structure of the core complex SctRST was obtained, in both the flagellar [32] and the T3SS [33] orthologues, and demonstrate that they form an intimate complex, with the predicted transmembrane helix in fact being shown to be mostly buried within the complex (Figure 2a), in a helical arrangement reminding of the needle. A subsequent structure of the flagellar complex included the SctU orthologue, adding an additional helical component to this pseudo-helix; nonetheless, the density of its soluble domain was not resolved, suggesting that it may be flexible, at least in the context of this isolated complex.

More recently, the above-mentioned cryo-EM structure of the intact NC included the export apparatus embedded within [28]. This structure largely confirmed its features (although no density for SctU was visible), but also provided the molecular basis for the junction between the export apparatus and the basal body, with notably revealing that an inner rod region, consisting of multiple copies of the protein SctI, in an arrangement resembling that of the filament, provided a direct junction between the helical arrangement of the export apparatus, and the needle. Only recently, lipids were found at defined positions within the needle complex, suggesting a crucial role for the assembly of functional T3SSs and establishing that the NC is in fact a protein-lipid complex [34]. In particular, lipids reside in the upper part of the export apparatus to stabilize the helical fork of the inner rod protein (PrgJ), which establishes the transition from the pseudohelical architecture of the export apparatus to the helical needle filament.

One additional protein, SctV, is closely associated to the export apparatus, but also possesses a large globular domain, located in the cytosol, whose role is to select effector proteins and target them for secretion, forming a so-called “sorting platform.” An early crystal structure of the globular domain on its own suggested that it forms a nonameric complex [35], an arrangement that was recently confirmed by cryo-EM studies of the intact protein [36,37]. However, in those structures, the transmembrane helices could not be resolved, and the relative position of SctV to that of the NC could not be determined. Evidence for this came from cryo-electron tomography studies of the prototypical *Salmonella* T3SS complex *in situ* [38], where clear density for SctV could be observed below the NC complex (Figure 2b). In spite of the relatively low resolution of the maps obtained by this method, the data demonstrated that this density consists of a nonameric complex of SctV, linked to the export apparatus.

Finally, an ATPase, SctN, with homology to the F1–V1 ATPase family [39], is associated with the T3SS, and was initially thought to provide the energy of protein translocation. However, biochemical evidence indicate a more complex mechanism: ATP hydrolysis is required for the initial step of substrate secretion, but additional force is provided by the proton motor force [40], via the TM region of SctV, which is a proton channel [41]. The most recent model suggests that the role of SctN is primarily to unfold the effector proteins, and dissociate them from their respective chaperones [42,43]. A cryo-EM structure of SctN, in its hexameric state and in complex with the associated “central stalk” protein SctO, was reported recently [44]. As above, its relative location to the T3SS NC was determined *in situ* by cryo-electron tomography [38], which demonstrated the presence of a complex of similar size ∼ 10 nm below the rest of the apparatus (Figure 2b). Intriguingly, it is surrounded by six pockets of density, that connect it to the NC. The nature of this density is not yet fully established, but they have been proposed to consist of the proteins SctK, SctL, and SctQ. The structural basis for the interaction between the ATPase and the NC remains to be characterized.

c) The needle tip and translocon

On the extracellular side of the complex, the needle is topped by a tip complex (Figure 2a), consisting of five copies of the protein SctA. While our molecular understanding had long remained limited to a low-resolution cryo-EM structure [45], combined with atomic models of isolated fragments [46, 47, 48, 49], a recent cryo-EM structure of this complex bound to the filament has revealed the details of its architecture, and the molecular basis of its interaction with the needle [50]. It is worth noting that the symmetry mismatch between the pentameric tip complex and the 11-stranded needle filament is similar to that of the bacterial flagellum tip complex [51], but the individual components do not share any sequence or structural homology.

Upon contact of the filament tip with a mammalian cell, a complex assembles at the tip, and forms a pore inside the target cell membrane. This so-called “translocon” pore consists of two proteins, the so-called minor and major translocon proteins SctE and SctB, but their structure and stoichiometry is not understood (Figure 2a). Most, but not all, translocon proteins have predicted transmembrane helices, however only a few crystal structures of soluble fragments have been reported to date [52,53], and do not seem to share any structural homology, even between them.

Recent low-resolution cryo-electron tomography data of T3SS complexes bound to mammalian cells has confirmed the formation of a pore docked to the tip complex [54,55], but the structural basis of this pore or their interaction with the tip is not known.

## Mechanism of T3SS secretion

As mentioned above, both injectisome and flagellar T3SSs contain a common and conserved so-called export apparatus, arranged as a pseudohelical, conical architecture within the cylindrical needle complex. This apparatus is positioned and stabilized by the ring-arranged proteins at the level of the inner membrane. The stoichiometry of the three export apparatus proteins SctR:S:T is 5:4:1, and represents the connection between the bacterial cytoplasm and the outer regions of the bacterial cell [32,33]. Although the export apparatus has an entrance to the presumed secretion channel at the cytoplasmic side and connects to the needle filament at the level of the periplasm, no continuous secretion channel could be observed from all known structures of the export apparatus: All structures contain a sealed gasket with an above loop suggesting that molecular changes are necessary for the transport of substrates.

Consequently, the most recent work has focused on the study of active T3SS and investigated them both biochemically and structurally using cryo-EM. However, a prerequisite for performing these studies, was to re-design the system such that substrates, whose transport usually proceeds at high rates, are stalled during transport.

**Figure 3:**
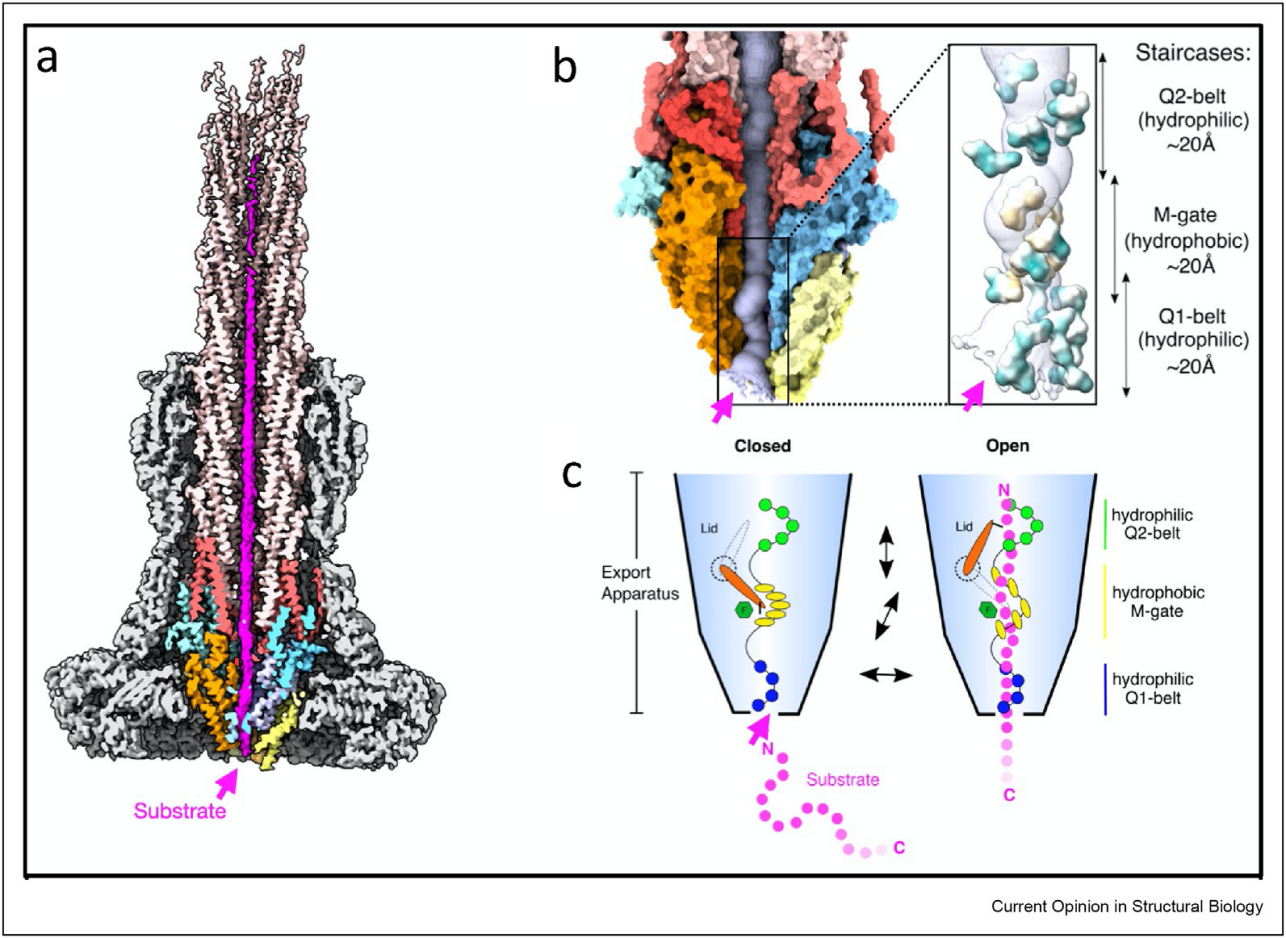
**Mechanism of effector transport through the T3SS. (a)** Cryo-EM map of the NC, with a substrate engaged. The density corresponding to the export apparatus is in yellow, orange and cyan, and the substrate is in magenta. **(b)** Close-up view of the portion of the translocation channel within the export gate. The Q1-, M− and Q2-gates are shown. **(c)** Schematic representation of the substrate export mechanism.

Such a novel substrate design included a secretion-active part fused by a part, whose protein domain cannot be unfolded by the activity of the T3SS [56]. These substrates thus remain in their transport form in the secretory channel and can be studied in detail in their new environment. Structural biological studies of active T3SSs using cryo-EM have now made it possible both to determine the structure of substrates within the secretion channel, and to study the export apparatus in its active state in more detail [34]. For the first time, it could be shown how and in which form an unfolded protein winds along the entire and open secretion channel (Figure 3a).

The export apparatus contains three prominent and discrete sections each about 20 Å in length, which form a three-point pseudo-helical interface with the substrate: the hydrophilic Q1-belt, followed by the hydrophobic M-gate, and complemented by another hydrophilic Q2-belt (Figure 3b). Substrates engage with and enter the channel with their N-terminus through the Q1 belt, through a side-chain independent manner explaining the plasticity of N-terminal signal sequences present in T3 effectors. Subsequently, the substrates pass through a disrupted methionine network leading to an opening of the M-gate (Figure 3c). Interestingly, only subtle conformational changes are required to disrupt this network, suggesting an elegant mechanism for opening and closing a small gate sufficient for transport of an unfolded polypeptide and preventing leakage of small molecules and nutrients. The importance of this gate has also been observed in flagellar T3SSs [57]. The SctT loop, which in the apo-complex rests above the M-gate and may support its tight closure, extends upwards and thereby generate a continuous secretion channel for the substrate. Moreover, in its upwards configuration, the R-loop reaches into the Q2-belt to adopt a conformation that allows guiding a passing substrate further up into the atrium and subsequently the lumen of the needle filament. Noteworthy, the cross sections of both the atrium and the tunnel of the filament are larger than 10 Å, allowing in principle substrates to be transported also as alpha helical segments. In contrast, the constriction at the open M-gate measures approximately 5.5 Å, requiring substrates to be completely unfolded during transport at that position and suggests the possibility that the highly constricted parts of the secretion tunnel within the export apparatus may also serve as a checkpoint to restrict access of only completely unfolded proteins.

## Conclusion: lessons learned and future challenges in cryo-EM studies of the T3SS

The past five years have seen a remarkable advance in our understanding of the T3SS structure, thanks mostly to the “resolution revolution” in cryo-EM. Nonetheless, several elements still elude our understanding:

- The translocon remains very poorly understood at the molecular level. The lack of apparent structural similarity between species presents a unique challenge, probably indicating that we are lacking a central information that would allow to elucidate this structure.
- Similarly, on the cytosolic side, the structure that links the ATPase to the export apparatus remains limited to low-resolution insights from tomography, and further work is necessary to characterize their interplay.
- Finally, the current structures of the T3SS do not fully explain how the SctN ATPase and SctV proton channel provide the energy to transport proteins through such a long distance.

Beyond this, while cryo-EM has provided snapshots of many structural aspects of the T3SS structure, the molecular details of the dynamics, and assembly, of this highly sophisticated nano-machine, mostly remains to be characterized. This includes in particular, the identification and recruitment of substrates on the cytosolic end [58], the establishment of various components fitting together in spite of having distinct symmetries [28], and the sensing of membrane attachment that triggers the secretion of effectors [59]. It is likely that a combination of structural, biochemical, and biophysical approaches will be necessary to fully understand those processes.

Ultimately, the “gold-standard” end-point will be to obtain high-resolution structural information of these complexes *in-situ*, by cryo-electron tomography. This would likely allow to resolve assembly intermediates, dynamic properties, and other aspects that cannot be captured by single-molecule approaches. In particular, the recent *in-situ* studies have revealed a range of structural changes to the T3SS upon contact with the target cells, but the resolution is too low to interpret these. In spite of very impressive advances in data processing in recent years [5], this is not currently feasible to obtain near-atomic resolution by tomography with the existing technology. It is likely that a second “resolution revolution” for tomography will be necessary to fully comprehend the molecular details of the T3SS.

## Conflict of interest statement

Nothing declared.
